# Cost-utility analysis of pralatrexate for relapsed or refractory peripheral T-cell lymphoma based on a case-matched historical control study along with single arm clinical trial

**DOI:** 10.1186/s12885-020-07629-z

**Published:** 2020-11-26

**Authors:** Seonyoung Park, Ah-Young Kim, Hyeonseok Cho, Deborah Baik, Hankil Lee, Sunghwa Cho, Hye-Young Kang

**Affiliations:** 1 Department of Pharmaceutical Medicine and Regulatory Sciences, Colleges of Medicine and Pharmacy, Yonsei University, Incheon, South Korea; 2 College of Pharmacy, Yonsei Institute of Pharmaceutical Sciences, Yonsei University, Incheon, South Korea; 3 Graduate School of Public Health, Yonsei University, Seoul, South Korea; 4 Institute of Health Services Research, Yonsei University, Seoul, South Korea

**Keywords:** Case-matched control analysis, Cost-utility analysis, Peripheral T-cell lymphoma, Pralatrexate

## Abstract

**Background:**

Patients with relapsed or refractory peripheral T-cell lymphoma (R/R PTCL) treated with pralatrexate have previously shown superior overall survival (OS) compared to those who underwent conventional chemotherapy (CC, 15.4 vs. 4.07 months). We conducted an economic evaluation of pralatrexate from a societal perspective in Korea based on data from the PROPEL phase II study.

**Methods:**

Using a Markov model with a weekly cycle, we simulated the experience of patients with R/R PTCL receiving pralatrexate or CC for 15 years. The model consists of five health states; initial treatment, treatment pause, subsequent treatment, stem cell transplantation (SCT) success, and death. Comparative effectiveness was based on PROPEL phase II single-arm study and its matched historical control analysis. Costs included drug, drug administration, monitoring, adverse event management, and SCT costs.

**Results:**

The incremental cost-effectiveness ratio of the base case was $39,153 per quality-adjusted life-year (QALY) gained. The results of one-way sensitivity analysis ranged from $33,949 to $51,846 per QALY gained, which remained within an implicit willingness-to-pay (WTP) threshold of anticancer drugs in Korea.

**Conclusions:**

Pralatrexate is a cost-effective intervention with improved OS and incremental costs within the WTP limit. Pralatrexate could function as a new therapeutic option for patients suffering from life-threatening R/R PTCL.

**Supplementary Information:**

The online version contains supplementary material available at 10.1186/s12885-020-07629-z.

## Background

Peripheral T-cell lymphoma (PTCL) is an aggressive and heterogeneous type of lymphoma that constitutes 7% of non-Hodgkin lymphoma (NHL) [1]. Although the incidence of PTCL is low (< 1 case per 100,000 population in the USA) [2], it has been increasing, in part due to advances in diagnosis methods as well as due to the aging population [3]. In contrast to other NHLs, PTCL has poor treatment outcomes with a five-year survival rate of 32% [4]. Approximately 68% of patients with PTCL are relapsed or refractory (R/R) to conventional chemotherapy (CC) [5]. Prognosis is more unfavorable for R/R PTCL, and no standard care exists. Median overall survival (OS) is 5.8 months, with three-year OS rates of less than 30% [5].

Pralatrexate is a single antifolate agent. By penetrating cancer cells, pralatrexate inhibits dihydrofolate reductase, thereby depleting the levels of biological molecules requiring thymidine or single carbon transfer, eventually causing cell death. Since pralatrexate is administered in an outpatient setting, it is more convenient than CC, which requires hospitalization for consecutive injections. The clinical outcomes of pralatrexate (OS: 14.5 months) were investigated by a phase II, single-arm, open label, international multicenter study (PROPEL study) on 111 patients with R/R PTCL [6]. Although the PROPEL study is the largest prospective study, it does not provide the comparative effectiveness of the drug. Therefore, as a follow-up study of PROPEL, a 1:1 case-matched control analysis (CMCA) of pralatrexate to CC was conducted using propensity score matching. Pralatrexate has shown superior clinical outcomes in terms of OS compared to CC, with significant improvement from 4.07 to 15.24 months [7].

Although pralatrexate use is advantageous due to improved OS, the increasing cost may prevent it from being widely accepted. Therefore, we assessed the cost-effectiveness of pralatrexate to better understand both the clinical and economic value of pralatrexate in the treatment of patient with R/R PTCL in Korea based on data from the PROPEL phase II study.

## Methods

### Model structure

**Fig. 1:**
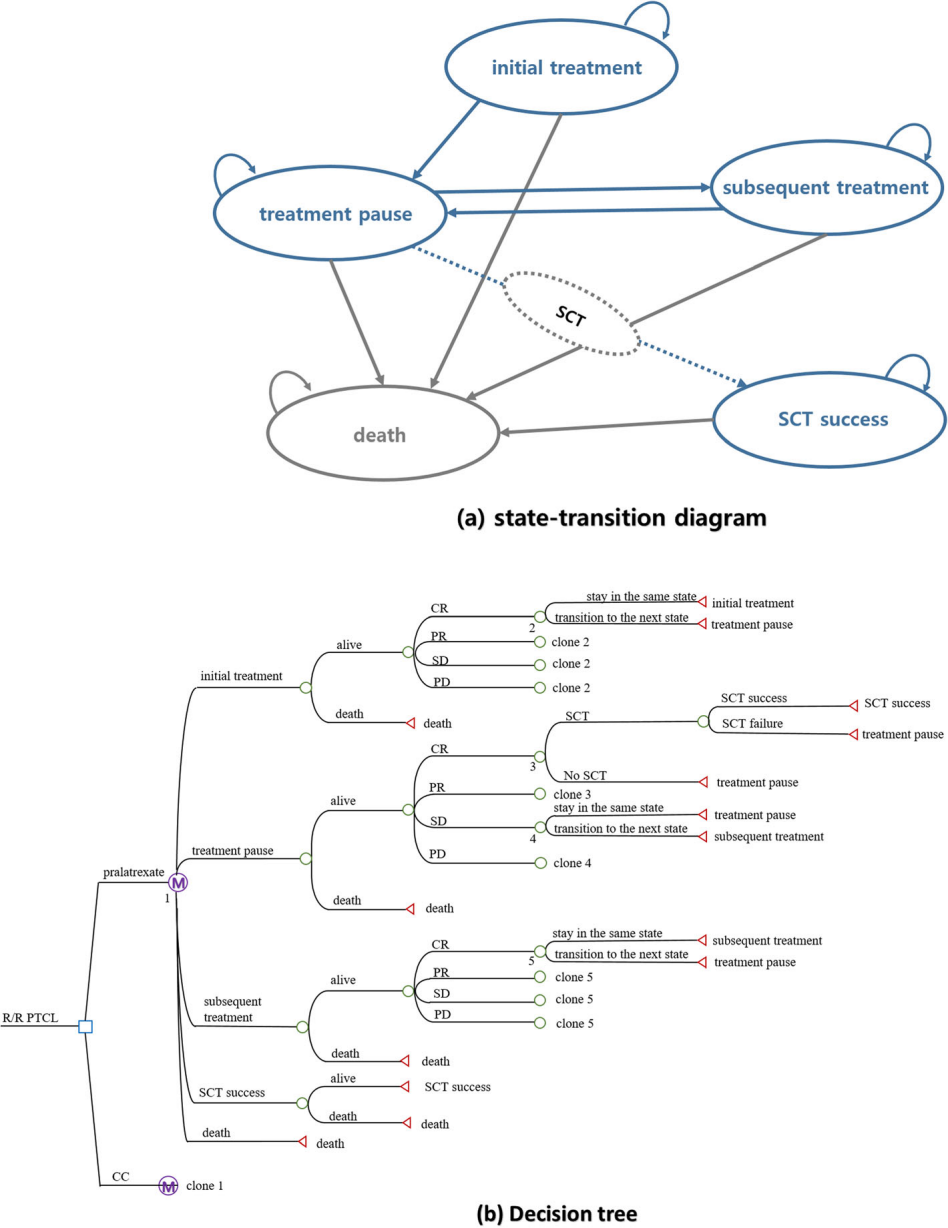
Structure of Markov model to assess cost-effectiveness of pralatrexate in treating R/R PTCL. (**a**) corresponds to state-transition diagram and (**b**) corresponds to decision tree. CC, conventional chemotherapy; CR, complete response; PD, progressive disease; PR, partial response; PTCL, peripheral T-cell lymphoma; R/R, relapsed or refractory; SCT, stem cell transplantation; SD, stable disease.

Using a Markov cohort model, the experience of patients with R/R PTCL receiving pralatrexate (treatment arm) or CC (comparator arm) was simulated. The cycle length of model, in which a health state transition can occur, was set as a week. The simulation began at 48 years of age, reflecting the mean age of Korean patients with R/R PTCL [8], and continued for 15 years. The model consisted of five health states; initial treatment, treatment pause, subsequent treatment, stem cell transplantation (SCT) success, and death (Fig. 1). All four alive health states can transition to death throughout the simulation. Except “SCT success” and “death” states, each health state was composed of four nested states; complete response (CR), partial response (PR), stable disease (SD), and progressive disease (PD). According to the PROPEL study, the proportion of patients achieving CR, PR, SD, or PD as a result of initial treatment with pralatrexate is 12.6, 21.2, 22.1, and 44.2%, respectively. Among the Korean patients included in the CMCA study [7], 19.8, 21.4, 3.2%, or 55.6% achieve CR, PR, SD, or PD following initial treatment with CC, respectively.

Patients from each arm began the simulation at the “initial treatment” state. It was assumed that the initial treatment state lasts for 14 weeks because the drug regimen for pralatrexate and CC is two treatment cycles with a seven-week cycle [6] and 4.67 cycles with a three-week cycle (according to a local clinician survey), respectively.

After completing the initial treatment, patients moved to the “treatment pause” stage, if they survived, for 8 weeks until subsequent treatment began. It was assumed that the response rate during the treatment pause state was the same as that during the initial treatment state. Among patients achieving CR or PR, those receiving SCT with a successful outcome moved to the “SCT success state” and it was presumed that they will have the same survival rate and quality of life as the general population. Therefore, the age-specific mortality rate of the general population provided by the Korean National Statistics Office was applied to them. Regardless of the type of drug treatments provided, the probability of receiving SCT in the treatment pause state following initial treatment was assumed to be 0.529 based on an expert panel survey of six clinicians who treat most R/R PTCL patients in Korea. The probability of success among those receiving SCT, defined as survival for at least 1 year, was 0.663 based on a local study [9]. In general, patients with R/R PTCL receive SCT when they achieve the best response [1]. According to the clinician panel survey, the median time of Korean patients with R/R PTCL achieving the best response is 20 weeks following initial treatment. Therefore, we assumed that SCT is performed at the 20th week from the start of initial treatment. Patients with CR not receiving SCT or receiving SCT with failure remained in the “treatment pause state” for the rest of the model simulation. The other patients (i.e., patients with PR not receiving SCT or receiving SCT with failure, and patients with SD or PD) transitioned to the “subsequent treatment” state at the 23rd week from the start of model simulation.

In the subsequent treatment state, all patients from either the treatment or comparator arm received CC for 4.33 cycles with a three-week cycle (according to a local clinician survey). After completing subsequent therapy, patients moved to the “treatment pause” state again on the 36th week. Among the Korean patients included in the CMCA study [7], the proportion of patients achieving CR, PR, SD, or PD following subsequent treatment was 12.3, 15.8, 3.5%, or 68.4%, respectively. Among patients with CR or PR in the treatment pause state, those receiving SCT with successful outcomes moved to the “SCT success state.” The probability of receiving SCT in the treatment pause state following subsequent treatment was assumed to be 0.458 based on the local clinician survey. The same probability of SCT success (0.663) was used. Applying local practice patterns, it was assumed that patients receive SCT at the 42nd week, which is 20 weeks after the subsequent therapy begins. The other patients (i.e., patients with CR or PR not receiving SCT or receiving SCT with failure, and patients with SD or PD) remained in the “treatment pause state” for the rest of the model simulation.

### Comparators

Patients assigned to the comparator arm were assumed to receive CC, such as DHAP (dexamethasone, cisplatin, and cytarabine), ESHAP (etoposide, methylprednisolone, cytarabine, and cisplatin), or ICE (ifosfamide, carboplatin, and etoposide), according to recommendations by the National Comprehensive Cancer Network guidelines and reimbursement list of the Korean National Health Insurance (NHI). The proportion of R/R PTCL patients receiving DHAP, ESHAP, or ICE in Korea was obtained from a clinician panel survey.

### Input parameter and data sources

Table 1 presents model parameters and their data sources.

1. Comparative effectiveness

**Table 1:** Model input parameters and data sources*

Parameters	Base-case values	Data source
Number of treatment cycles		
Initial treatment: pralatrexate	2 cycles with a 7-week cycle	PROPEL study [6]
Initial tx.: CC	4.67 cycles with a 3-week cycle	Clinician panel survey
Subsequent tx.: CC	4.33 cycles with a 3-week cycle	Clinician panel survey
Transition probability		
Type of distribution used to extrapolate survival curves		
Pralatrexate	Generalized gamma	O’Connor et al. [7]
CC	Gompertz	O’Connor et al. [7]
Response rate		
Initial treatment: pralatrexate		PROPEL study [6]
CR	0.126	
PR	0.211	
SD	0.221	
PD	0.442	
Initial tx.: CC		Estimation based on
CR	0.198	Korean subjects [7]
PR	0.214	
SD	0.032	
PD	0.556	
Subsequent tx.: CC		Estimation based on
CR	0.123	Korean subjects [7]
PR	0.158	
SD	0.035	
PD	0.684	
SCT rate following initial tx.	0.529	Clinician panel survey
SCT success rate following initial tx.	0.663	Kim et al. [9]
SCT rate following subsequent tx.	0.458	Clinician panel survey
SCT success rate following subseq. tx.	0.663	Kim et al. [9]
Probability of AE		
Mucositis (ICD-10 codes: K123)		PROPEL study [6]
Initial tx.: pralatrexate	0.0172	Crump et al. [10];
Initial tx.: CC	0.0365	Jerkeman et al. [11];
Subsequent tx.: CC	0.0393	Wang et al. [12];
Thrombocytopenia (D695)		Velasquez et al. [13];
Initial tx.: pralatrexate	0.0267	Ezzat et al. [14];
Initial tx.: CC	0.1122	Press et al. [15]
Subsequent tx.: CC	0.0839	
Anemia (D611, D630)		
Initial tx.: pralatrexate	0.0118	
Initial tx.: CC	0.0257	
Subsequent tx.: CC	0.0279	
Neutropenia (D70)		
Initial tx.: pralatrexate	0.0172	
Initial tx.: conventional chemo.	0.1250	
Subsequent tx.: conventional chemo.	0.1011	
Nausea/vomiting (R11)		
Initial tx.: pralatrexate	0.0026	
Initial tx.: conventional chemo.	0.0053	
Subsequent tx.: conventional chemo.	0.0070	
Peripheral neuropathy (G900)		
Initial tx.: pralatrexate	0.0000	
Initial tx.: conventional chemo.	0.0063	
Subsequent tx.: conventional chemo.	0.0053	
Costs^1^ (US dollars)		
Medication cost^2^		
Initial tx.: pralatrexate	$2465	Manufacturer’s suggesting price
Initial tx.: CC	$240	Maximum Payable Amount Table of
Subseq. tx.: CC	$238	
Concomitant drug cost		Korean NHI, Clinician panel survey
Initial tx.: pralatrexate		
First cycle, last cycle	$4, $3	
Initial tx.: CC	$207	
Subseq. tx.: CC	$198	
SCT cost	$27,343	HIRA-NPS data (2011–2016), KHSCTA, KMDP
Routine monitoring test cost		Maximum Payable Amount Table of
Initial tx.: pralatrexate		
First cycle, other cycles	$106, $17	Korean NHI, Clinician panel survey
Initial tx.: CC		
First cycle, other cycles	$129, $40	
Subseq. tx.: CC		
First cycle, other cycles	$123, $38	
Costs to treat AE^3^		Korean NHI healthcare statistics
Mucositis	$1288	
Thrombocytopenia	$2859	
Anemia	$2877	
Neutropenia	$2760	
Nausea/vomiting	$707	
Peripheral neuropathy	$643	
Utility (disutility)		
CR	0.885	Kang et al. [16]
PR	0.784	
SD	0.746	
PD	0.567	
SCT success^4^: 45–49, 50–54, 55–59, 60–64 years old	0.976, 0.971, 0.966, 0.936	KNHANES (2014)
Mucositis	(−0.075)	Kang et al. [16]
Thrombocytopenia	(−0.095)	Kang et al. [16]
Anemia	(−0.085)	Kang et al. [16]
Neutropenia	(− 0.107)	Kang et al. [16]
Nausea/vomiting	(− 0.059)	Nafees et al. [17]
Peripheral neuropathy	(−0.42)	Swinburn et al. [18]

*AE* Adverse event; *CC* Conventional chemotherapy; *CR* Complete response; *HIRA-NPS* Health Insurance Review and Assessment Service-National Patients Sample; *ICD-10 code* International Classification of Disease code 10th edition; *KHSCTA* Korean Hematopoietic Stem Cell Transplantation Association; *KMDP* Korea Marrow Donor Program; *KNHANES* Korea National Health and Nutrition Examination Survey; *NHI* National Health Insurance; *PD* Progressive disease; *PR* Partial response; SD Stable disease; *SCT* Stem cell transplantation

^1.^ All costs are presented as weekly costs in 2019 US dollars and were estimated from a societal perspective, including medical costs, transportation costs to visit health care institutions, and care giver’s costs for hospitalization. ^2.^ Medication costs include drug costs and drug administration costs, such as costs of outpatient visits, hospitalization, medication management, aseptic preparation, and injections. ^3.^ Since all the adverse events (AEs) included are grade 3 or higher and require hospitalization, the cost to treat each AE was measured as the average cost per hospitalization with the condition. ^4.^ Utility during the post-SCT health states (i.e., SCT success state) was assumed to be the same as that of the general population, which is the average utility value for the general population aged 45 to 65 years obtained from 2014 KNHANES data

The comparative effectiveness of pralatrexate versus CC was derived from a CMCA study [7]. Patients treated with pralatrexate had a median OS of 15.24 months, while those in the control group had 4.07 months (hazard ratio (HR): 0.432, 95% confidence interval (CI): 0.298 to 0.626).

**Fig. 2:**
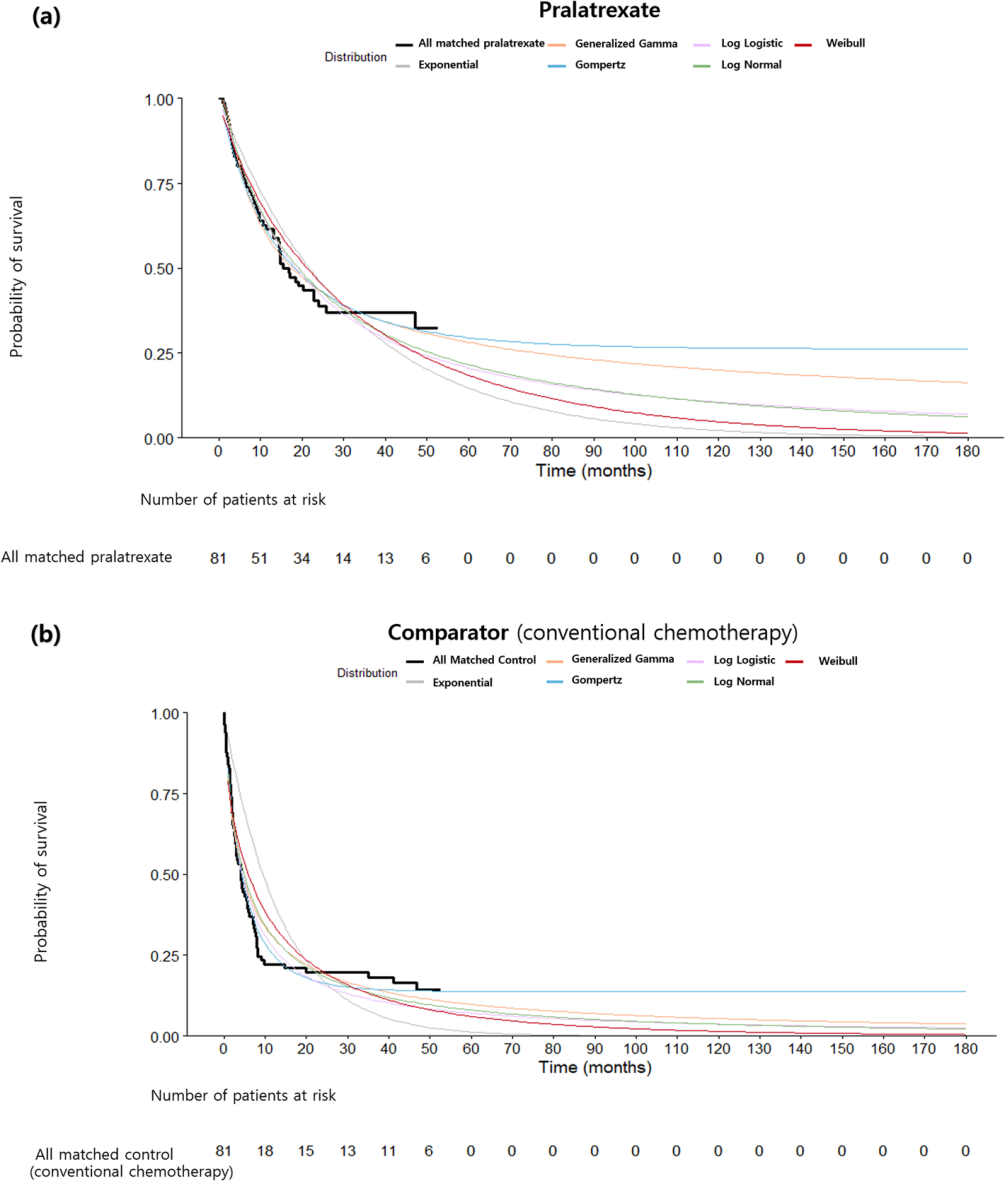
Kaplan-Meier and parametric survival curves of overall survival. (**a**) corresponds to patients treated with pralatrexate and (**b**) corresponds to patients treated with conventional chemotherapy

Transition probabilities were derived from the CMCA study by digitizing OS rates from Kaplan-Meier curves. Since the time horizon for the cost-effectiveness analysis was greater than the duration of the CMCA study (approximately 52 months), extrapolation of survival rates was required to provide the long-term survival benefits of pralatrexate. Since access to individual patient data (IPD) was not possible, it was reconstructed from the CMCA study, so that parametric survival analysis could be conducted. The Kaplan-Meier survival curves for both treatment groups were digitized using DigitizeIt software. The method developed by Guyot et al. was applied to reconstruct IPD from the extracted coordinates of the published Kaplan-Meier survival curves using R software [19]. The estimated median OS and HR were similar to those of the CMCA study, demonstrating the validity of the reconstructed data [20]. Parametric survival analyses were conducted corresponding to guidance from the National Institute for Health and Care Excellence [21]. The proportional hazards assumption did not hold according to goodness-of-fit test (*p*-value < 0.05), indicating that fitting parametric models separately to each treatment arm may be preferred. Parametric survival curves were fitted to the reconstructed IPD using various distribution functions. This approach was based on the NICE technical support document 14: survival analysis for economic evaluations alongside clinical trials-extrapolation with patient-level data, which says “There are a wide range of parametric models available, and each have their own characteristics which make them suitable for different data sets. Exponential, Weibull, Gompertz, log-logistic, log normal and Generalised Gamma parametric models should all be considered.” [22]. In order to identify the most appropriate distribution function, Akaike Information Criterion (AIC) and Bayesian Information Criterion (BIC) as well as visual inspection were assessed. For the pralatrexate arm, a generalized gamma model was selected as the best fit by visual inspection, as well as the lowest AIC(434.1) and BIC(431.4) (Fig. 2). In contrast, the Gompertz model was selected as the most appropriate for the comparator group (AIC: 436.3, BIC: 433.7) (Fig. 2). Transition probabilities of death beyond the duration of the CMCA study were then calculated from survival curves in each arm.

2 Costs

Costs were estimated from a societal perspective, including medical costs, transportation costs to visit healthcare institutions, and caregiver’s costs for hospitalization. All costs are presented as US dollars in 2019 value (1 USD approximately equal to 1100 Korean won).

#### Cost of initial treatment

Initial treatment is provided with pralatrexate or comparator drugs. For each treatment, we calculated drug costs, drug administration costs, costs to treat adverse events (AEs), and monitoring costs. The cost of the comparator arm was calculated as a weighted average based on the usage proportion of DHAP (15.3%), ESHAP (9.9%), and ICE (74.8%) obtained through clinical expert consultation.

Based on the unit price, and doses recommended by the NCCN guidelines, weekly drug costs were calculated as $2279 for pralatrexate, $54 for DHAP, $64 for ESHAP, and $100 for ICE. Drug costs of essential concomitant drugs were included: oral folic acid and vitamin B12 for pralatrexate, and pegfilgrastim, pegteograstim, tripegfilgrastim, and lipegfilgrastim for comparator drugs. Drug administration costs included outpatient visits, hospitalization, medication management, aseptic preparation, and injections. Unit costs for each item were determined using the Maximum Payable Amount Table of Korean NHI.

The common AEs among patients treated with pralatrexate or comparator drugs include anemia, mucositis, nausea/vomiting, neutropenia, peripheral neuropathy, and thrombocytopenia. The level of AE for all of these is grade 3 or higher, which indicates AEs requiring hospitalization [23]. Therefore, the average cost per hospitalization for each condition reported by the Korean NHI statistics was used as the cost of treatment for the AE. Probabilities of AEs in each arm were obtained from literature [6, 10–15], and applied during the initial and subsequent treatment state.

Type and frequency of monitoring tests routinely performed for patients with R/R PTCL were identified by a clinician panel survey; positron emission tomography-torso; computed tomography for chest, abdomen, and neck; complete blood cell count; and general chemical tests.

#### Cost of subsequent treatment

In the subsequent treatment state, patients with R/R PTCL from both arms receive CC and best supportive care (BSC). Drug costs, drug administration costs, costs to treat AEs, and monitoring costs were calculated as above. Medical costs of the BSC were derived from a local study [24]. Costs of subsequent treatments were calculated as a weighted average based on the usage proportion of DHAP (33.9%), ESAHP (16.2%), ICE (45.5%), and BSC (4.4%).

#### Cost of SCT

From Health Insurance Review and Assessment Service-National Patients Sample (HIRA-NPS) data from 2011 to 2016, patients with PTCL, defined as those having at least one claim record with a diagnosis of PTCL (ICD-10 codes: C84.4, C84.6, C84.7, C86.0 ~ C86.3, C86.5, C86. 6, or C91.5), hospitalized for hematopoietic stem cell injection (X5131 ~ X5136) were identified. The average hospitalization cost was considered as cost per autologous SCT. Unlike autologous SCT patients using their own stem cells, allogeneic SCT patients use hematopoietic stem cells from other healthy individuals. Therefore, cost per allogenic SCT was computed as cost per autologous SCT plus cost of obtaining matched hematopoietic stem cells, estimated using the data from the Korean Hematopoietic Stem Cell Transplantation Association and the Korea Marrow Donor Program. Using the proportion of autologous and allogeneic SCT performed among patients with PTCL in HIRA-NPS data from 2011 to 2016, the weighted average cost of SCT among patients with PTCL was calculated.

3 Quality of life

Utility values for each health state and disutility values for AEs were obtained from a local study [16] and foreign literature [17, 18]. Utility decrement due to AEs was applied during the initial and subsequent treatment state. Utility during the SCT success state was assumed to be the same as that of the general population aged 45 to 65 years [25].

### Data analysis

During the modeled simulation of 15 years from the beginning at the “initial treatment” state, the average cost that a patient with R/R PTCL treated with pralatrexate or CC would have, was calculated for each Markov cycle by multiplying the probability of transition to each of the five health states during the cycle and their corresponding costs, and calculating the sum of the costs of all five health states. Then, we calculated the sum of the average costs of all Markov cycles (i.e., from the first cycle (1st week) through the last cycle (782nd week (=365 days/7 days × 15 years) of the Markov model) for each treatment arm, considering them as the expected costs that a patient with R/R PTCL would have for 15 years from the start of the simulation. The expected treatment effectiveness, defined as life-years (LYs) or quality-adjusted life-years (QALYs) gained as a result of treatment with pralatrexate or CC, was estimated in the same manner as the costs. For each Markov cycle, the average LYs were calculated as a product of the “probability of transition to each of the five health states during the cycle” and “1/52.1 or 0 LYs (= “1 week/52.1 weeks” if alive or “0 week/52.1 weeks” if dead).” Detailed explanations for how the expected costs and treatment effectiveness were computed as a result of the modeled simulation are presented in Additional file 1.

Incremental cost-effectiveness ratios (ICERs) were computed for the base case by dividing the incremental costs associated with providing pralatrexate therapy versus CC (i.e. the expected costs of pralatrexate arm less the expected costs of CC arm) by incremental effectiveness measured in LYs and QALYs gained, respectively. An annual discounted rate of 5% was used for both cost and effectiveness.

One-way sensitivity analyses were conducted to confirm the robustness of the ICER of the base case. The impact of the uncertainty in the extrapolation of the OS benefit of pralatrexate beyond the observation period of the CMCA was examined by applying different distribution functions. For utility for the health states of CR, PR, SD, and PD, upper and lower values of 95% CI [16] were used to re-calculate the ICER. The success rate of SCT was varied by 10% from the base case. Assuming that dose intensity is correlated with survival gain in lymphoma [26], a 20% decrease was applied to the dose of comparator drugs due to AEs, and the corresponding costs were adjusted accordingly. Based on the time horizon used in the earlier studies, the time horizon of the modelled simulation was varied to 10 and 30 years.

## Results

In the base-case analysis, the expected cost per patient for 15 years in the simulation was $48,677 in the pralatrexate arm and $20,045 in the comparator arm, resulting in an incremental cost of $28,632. Patients in the pralatrexate arm incurred more initial treatment drug costs ($30,301), subsequent treatment costs (treatment drugs: $1033, concomitant drugs: $860, and monitoring tests: $179), and SCT costs ($1953) than patients in the comparator arm. Higher costs associated with subsequent treatment and SCT were due to improved OS in the pralatrexate arm. The incremental LYs and QALYs of patients in the pralatrexate arm compared with those in the comparator arm were 1.567 years (3.499 vs. 1.932 years) and 0.731 QALYs (1.075 vs. 0.344 QALYs), respectively. As a result, the ICER is estimated to be $18,276 per LY gained and $39,153 per QALY gained (Table 2).

**Table 2:** Base-case analysis results for cost-effectiveness of pralatrexate versus conventional chemotherapy in treating R/R PTCL^1^

	Pralatrexate	Conventional chemotherapy	Incremental value (pralatrexate vs. coven. Chemo.)
Total costs ($)	48,677	20,045	28,632
Initial treatment cost	32,804	2504	30,301
Concomitant drug cost (initial tx.)	45	2167	-2122
Subsequent treatment cost	1842	810	1033
Concomitant drug cost (subseq. tx.)	1533	674	860
Monitoring test cost (initial tx.)	273	461	− 188
Monitoring test cost (subseq. tx)	322	143	179
Cost to treat AE	7170	10,554	− 3383
SCT cost	4686	2733	1953
Life years (LYs)	3.499	1.932	1.567
Quality-adjusted life years (QALYs)	1.075	0.344	0.731
Incremental cost per LY gained	–	–	18,276
Incremental cost per QALY gained	–	–	39,153

*AE* Adverse event; *PTCL* Peripheral T-cell lymphoma; *R/R* Relapsed or refractory; *SCT* Stem cell transplantation

^1.^ All costs are presented as US dollars in 2019 value

**Fig. 3:**
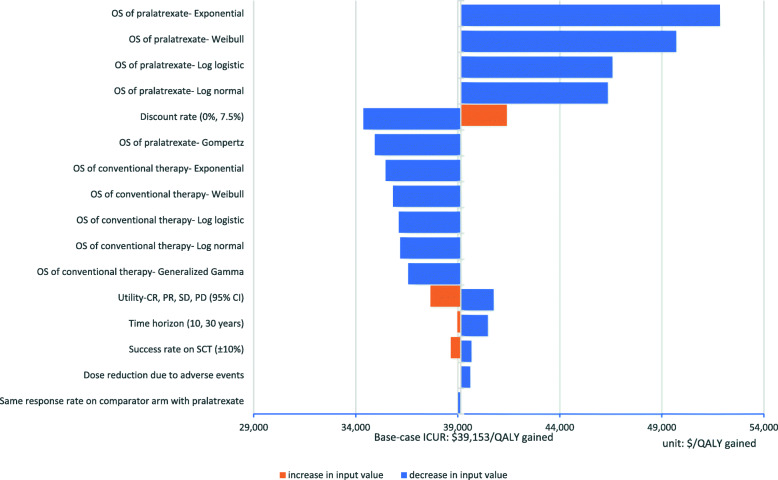
Tornado diagram for sensitivity analysis results**.** CR, complete remission; ICER, incremental cost-effectiveness ratio; PR, partial remission; SCT, stem cell transplantation; SD, stable disease; OS, overall survival; PD, progressive disease; USD, US dollars

Figure 3 presents the results of one-way sensitivity analysis. Overall, the ICER ranged from $33,949 to $51,846 per QALY gained. It remained within twice the per-capita gross domestic product (GDP, approximately $27,000) of Korea, which is an implicit willingness-to-pay (WTP) threshold of anticancer drugs to accept health benefit of one QALY gained in Korea. While the type of distribution function used to extrapolate survival curves was the most influential variable, resulting in a wide ICER range, the re-calculated ICERs remained within twice the GDP value. Therefore, we conclude that the base-case analysis result is robust to uncertainties of the model parameters.

## Discussion

Although pralatrexate proved its efficacy in the PROPEL study and received accelerated approval by the US Food and Drug Administration in 2009, there has been difficulty in obtaining reimbursement decisions by many other regulatory authorities due to the lack of comparative effectiveness data. Since the PROPEL study was a single arm study, it was not able to provide the merits of pralatrexate in comparison with conventional standards. To overcome this limitation, a CMCA of the PROPEL study population (80 patients) was conducted using OS data of propensity-score-matched historical controls (80 patients) from four academic groups on three continents [7]. Although a CMCA is not superior to randomized controlled trials (RCTs) in assessing the clinical benefits of a drug, propensity score matching allows an observational (non-randomized) study to mimic an RCT by making observed baseline covariates similar between the case and control groups [27, 28]. The provision of comparative effectiveness evidence of pralatrexate vs. CC enabled us to assess the relative economic value of pralatrexate.

Currently, pralatrexate is the only available monotherapy for patients with R/R PTCL. To the best of our knowledge, this is the first cost-effectiveness study of pralatrexate. The ICER for pralatrexate compared with CC yielded $39,153 per QALY gained, which is within the cost-effective threshold of Korea. Robust deterministic sensitivity analysis results confirmed that uncertainties of model parameters did not alter the cost-effective merit of pralatrexate.

Several assumptions and study limitations would over- or under-estimate the ICER. First, the success rate of SCT obtained from local literature [9] was based on the one-year survival rate, thus, only LY and QALY gain for those surviving for at least 1 year following SCT were reflected in our analysis. Those who survived for less than 1 year were not considered in the analysis. This results in underestimation of the clinical benefit of the improved OS of pralatrexate and, consequently, overestimation of the ICER. Second, we assumed that patients moving to the SCT success state have the same mortality rate and quality of life as the general population. This assumption excludes the possibility of recurrence or metastasis of cancer, leading to overestimation of the clinical benefits of pralatrexate and, consequently, underestimation of the ICER. Third, due to the lack of data for rare diseases, including R/R PTCL, it was not easy to find relevant data sources for model parameters. Therefore, we often depended on local clinician consultation. This could threaten the validity of our study results. However, the six clinicians who participated in the expert panel are highly representative as they treat most Korean R/R patients.

Nevertheless, this study has the strength of best utilizing local data. We incorporated real-world evidence in the modelled simulation by reflecting treatment patterns for patients with R/R PTCL based on the representative clinical expert’s consultation. All cost data were derived from various local sources, such as local unit cost, clinician panel survey, NHI statistics, and local patient dataset. Likewise, this study utilized local utility values for health states included in the model.

## Conclusions

Pralatrexate is a cost-effective intervention as it has a significant clinical benefit of improved OS and requires incremental costs within the WTP limit. We anticipate that pralatrexate could be a new therapeutic option for patients with life-threatening R/R PTCL.

## Supplementary Information

**Additional file 1.**

## Data Availability

All data generated and analyzed during this study are presented in Table 1 of this manuscript or available in the manuscript. Datasets are available through the corresponding author on reasonable request.

## Abbreviations

PTCL: Peripheral T-cell lymphoma
NHL: Non-hodgkin lymphoma
R/R: Relapsed or refractory
CC: Conventional chemotherapy
OS: Overall survival
CMCA: Case-matched control analysis
SCT: Stem cell transplantation
CR: Complete response
PR: Partial response
SD: Stable disease
PD: Progressive disease
DHAP: Dexamethasone, cisplatin, and cytarabine
ESHAP: Etoposide, methylprednisolone, cytarabine, and cisplatin
ICE: Ifosfamide, carboplatin, and etoposide
NHI: National health insurance
HR: Hazard ratio
CI: Confidence interval
IPD: Individual patient data
AIC: Akaike information criterion
BIC: Bayesian information criterion
AEs: Adverse events
BSC: Best supportive care
HIRA-NPS: Health insurance review and assessment service-national patients sample
ICER: Incremental cost-effectiveness ratio
LYs: Life years
QALYs: Quality-adjusted life-years
WTP: Willingness-to-pay.

## References

[1] Beaven AW, Diehl LF (2015). Peripheral T-cell lymphoma, NOS, and anaplastic large cell lymphoma. ASH Educ Program Book.

[2] Morton L, Wang SS, Devesa SS (2006). Lymphoma incidence patterns by WHO subtype in the United States, 1992−2001. Blood.

[3] Zing N, Fischer T, Zain J (2018). Peripheral T-cell lymphomas: incorporating new developments in diagnostics, prognostication, and treatment into clinical practice—PART 1: PTCL-NOS, FTCL, AITL, ALCL. Oncology.

[4] Federico M, Bellei M, Marcheselli L (2018). Peripheral T cell lymphoma, not otherwise specified (PTCL-NOS). A new prognostic model developed by the international T cell project network. Br J Haematol..

[5] Bellei M, Foss FM, Shustov AR (2018). The outcome of peripheral T-cell lymphoma patients failing first-line therapy: a report from the prospective, International T-cell project. Haematologica.

[6] O’Connor OA, Pro B, Pinter-Brown L (2011). Pralatrexate in patients with relapsed or refractory peripheral T-cell lymphoma: results from the pivotal PROPEL study. J Clin Oncol.

[7] O’Connor OA, Marchi E, Volinn W (2018). Strategy for assessing new drug value in orphan diseases: an international case match control analysis of the PROPEL study. JNCI Cancer Spectr.

[8] Kim JM, Ko YH, Lee SS (2011). WHO classification of malignant lymphomas in Korea: report of the third nationwide study. Korean J Pathol.

[9] Kim SW, Yoon SS, Suzuki R (2009). Autologous versus allogeneic hematopoietic stem cell transplantation (SCT) for peripheral T-cell lymphomas (PTCLs): Japan and Korea cooperative study with 330 patients. Blood.

[10] Crump M, Kuruvilla J, Couban S (2014). Randomized comparison of gemcitabine, dexamethasone, and cisplatin versus dexamethasone, cytarabine, and cisplatin chemotherapy before autologous stem-cell transplantation for relapsed and refractory aggressive lymphomas: NCIC-CTG LY. 12. J Clin Oncol.

[11] Jerkeman M, Leppa S, Kvaloy S (2004). ICE (ifosfamide, carboplatin, etoposide) as second-line chemotherapy in relapsed or primary progressive aggressive lymphoma-the Nordic lymphoma group experience. Eur J Haematol.

[12] Wang WS, Chiou TJ, Liu JH (1999). ESHAP as salvage therapy for refractory non-Hodgkin's lymphoma: Taiwan experience. Jpn J Clin Oncol.

[13] Velasquez WS, Cabanillas F, Salvador P (1988). Effective salvage therapy for lymphoma with cisplatin in combination with high-dose Ara-C and dexamethasone (DHAP). Blood.

[14] Ezzat AA, Khalifa F, Berry J (1994). E-SHAP: an effective treatment in selected patients with relapsed non-Hodgkin's lymphoma. Ann Oncol.

[15] Press OW, Livingston R, Mortimer J (1991). Treatment of relapsed non-Hodgkin's lymphomas with dexamethasone, high-dose cytarabine, and cisplatin before marrow transplantation. J Clin Oncol.

[16] Kang HN, Choi I, Song H (2015). Measurement of health state utilities for relapsed or refractory peripheral T-cell lymphoma by using time trade-off and visual analog scale method. Value Health.

[17] Nafees B, Lloyd AJ, Dewilde S (2017). Health state utilities in non–small cell lung cancer: an international study. Asia Pac J Clin Oncol.

[18] Swinburn P, Shingler S, Acaster S (2015). Health utilities in relation to treatment response and adverse events in relapsed/refractory Hodgkin lymphoma and systemic anaplastic large cell lymphoma. Leuk Lymphoma.

[19] Guyot P, Ades AE, Ouwens MJ, Welton NJ (2012). Enhanced secondary analysis of survival data: reconstructing the data from published Kaplan-Meier survival curves. BMC Med Res Methodol.

[20] Cho H, Heo SJ, Kim A, Kang HY (2019). Reconstruction of individual patient data from published survival curves: case of pralatrexate for peripheral T-cell lymphomas. Value Health.

[21] Latimer NR (2013). Survival analysis for economic evaluations alongside clinical trials—extrapolation with patient-level data: inconsistencies, limitations, and a practical guide. Med Decis Mak.

[22] Latimer N. NICE DSU technical support document 14: survival analysis for economic evaluations alongside clinical trials-extrapolation with patient-level data. Sheffield: Report by the Decision Support Unit; 2013. 27905716

[23] U.S. Department of health and human services. National Institutes of Health. National Cancer Institute Common Terminology Criteria for Adverse Events (CTCAE) Version 5.0. 2017.

[24] Korean Ministry of Health and Welfare. National Health and Nutrition Examination Survey Report 2005. 2006.

[25] Korean Ministry of Health and Welfare. National Health and Nutrition Examination Survey Report 2014. 2015.

[26] Yamaguchi H, Hirakawa T, Inokuchi K (2011). Importance of relative dose intensity in chemotherapy for diffuse large B-cell lymphoma. J Clin Exp Hematop.

[27] Ross ME, Kreider AR, Huang YS (2015). Propensity score methods for analyzing observational data like randomized experiments: challenges and solutions for rare outcomes and exposures. Am J Epidemiol.

[28] Austin PC (2013). The use of propensity score methods with survival or time-to-event outcomes: reporting measures of effect similar to those used in randomized experiments. Stat Med.

