# Supplementary material for: Cost-utility analysis of pralatrexate for relapsed or refractory peripheral T-cell lymphoma based on a case-matched historical control study along with single arm clinical trial
Source: BMC Cancer. 2020 Nov 26;20:1157. doi: 10.1186/s12885-020-07629-z (PMC7690091; doi:10.1186/s12885-020-07629-z)
Supplement: Supplementary file 1 — Additional file 1. [file 12885_2020_7629_MOESM1_ESM.docx]

**Appendix 1. Methods of computing the expected costs and treatment effectiveness (life-years or quality-adjusted life-years gained) for a patient with R/R PTCL treated with pralatrexate or conventional chemotherapy during 15 years of the modeled simulation**

Expected cost (PT) = $\sum_{j=1}^{n782} \sum_{i=1}^{5} PROBij\left( PT \right)xCOSTij(PT)$

Where,

Expected cost (PT) = the expected cost that a *pralatrexate*-treated patient with R/R PTCL would have for 15 years from the start of the simulation

i = 1 if “initial treatment state,” 2 if “treatment pause state,” 3 if “subsequent treatment state,” 4 if “SCT success state,” and 5 if “dead state”

j = 1^st^,…, 782^nd^ cycle

PROB_ij_ (PT) = probability of transition to the health state “i” during the cycle “j” for a *pralatrexate*-treated patient with R/R PTCL

COST_ij_ (PT) = cost associated with the health state “i” during the cycle “j” for a *pralatrexate*-treated patient with R/R PTCL

Expected cost (CC) = $\sum_{j=1}^{782} \sum_{i=1}^{5} PROBij\left( CC \right)xCOSTij(CC)$

where,

Expected cost (CC) = the expected cost that a *conventional chemotherapy*-treated patient with R/R PTCL would have for 15 years from the start of the simulation

PROB_ij_ (CC) = probability of transition to the health state “i” during the cycle “j” for a *conventional chemotherapy*-treated patient with R/R PTCL

COST_ij_ (CC) = cost associated with the health state “i” during the cycle “j” for a *conventional chemotherapy*-treated patient with R/R PTCL

Expected LYs (PT) = $\sum_{j=1}^{782} \sum_{i=1}^{5} PROBij\left( PT \right)xUnitLYs$

Where,

Expected LYs (PT) = the expected life-years that a *pralatrexate*-treated patient with R/R PTCL would have for 15 years from the start of the simulation

Unit LYs = “1 week/52.1 weeks” if alive or “0 week/52.1 weeks” if dead

Expected LYs (CC) = $\sum_{j=1}^{782} \sum_{i=1}^{5} PROBij\left( CC \right)xUnitLYs$

where,

Expected LYs (CC) = the expected life-years that a *conventional chemotherapy*-treated patient with R/R PTCL would have for 15 years from the start of the simulation

Expected QALYs (PT) = $\sum_{j=1}^{782} \sum_{i=1}^{5} PROBij\left( PT \right)x$UnitLYs x Uij(PT)

where,

Expected QALYs (PT) = the expected quality-adjusted life-years that a *pralatrexate*-treated patient with R/R PTCL would have for 15 years from the start of the simulation

U_ij_ (PT) = utility weight of being in the health state “i” during the cycle “j” for a *pralatrexate*-treated patient with R/R PTCL

Expected QALYs (CC) = $\sum_{j=1}^{782} \sum_{i=1}^{5} PROBij\left( CC \right)x$UnitLYs x Uij(CC)

Where,

Expected QALYs (CC) = the expected quality-adjusted life-years that a *conventional chemotherapy*-treated patient with R/R PTCL would have for 15 years from the start of the simulation

U_ij_ (CC) = utility weight of being in the health state “i” during the cycle “j” for a *conventional chemotherapy*-treated patient with R/R PTCL
